# TaSPL6B, a member of the Squamosa promoter binding protein-like family, regulates shoot branching and florescence in *Arabidopsis thaliana*

**DOI:** 10.1186/s12870-024-05429-2

**Published:** 2024-07-25

**Authors:** Feiyan Dong, Jinghan Song, Huadong Zhang, Jiarun Zhang, Yangfan Chen, Xiaoyi Zhou, Yaqian Li, Shijie Ge, Yike Liu

**Affiliations:** 1 Institute of Food Crops, Hubei Academy of Agricultural Sciences/ Key Laboratory of Crop Molecular Breeding, Ministry of Agriculture and Rural Affairs, Hubei Key Laboratory of Food Crop Germplasm and Genetic Improvement, Wuhan, 430064 China; 2 National Key Laboratory of Rice Biology, Institute of Crop Sciences, Zhejiang University, Hangzhou, 310058 China; 3 MARA Key Laboratory of Sustainable Crop Production in the Middle Reaches of the Yangtze River (Co- construction by Ministry and Province), College of Agriculture, Yangtze University, Jingzhou, 434025 China

**Keywords:** Wheat, Ectopic expression, *TaSPL6B*, Tillering, Light signaling pathway, Strigolactone signaling pathway

## Abstract

**Background:**

Squamosa promoter-binding protein-like (SPL) proteins are essential to plant growth and development as plant-specific transcription factors. However, the functions of SPL proteins in wheat need to be further explored.

**Results:**

We cloned and characterized TaSPL6B of wheat in this study. Analysis of physicochemical properties revealed that it contained 961 amino acids and had a molecular weight of 105 kDa. Full-length TaSPL6B transcription activity was not validated in yeast and subcellular localization analysis revealed that TaSPL6B was distributed in the nucleus. Ectopic expression of *TaSPL6B* in *Arabidopsis* led to increasing number of branches and early flowering. *TaSPL6B* was highly transcribed in internodes of transgenic *Arabidopsis*. The expression of *AtSMXL6/AtSMXL7/AtSMXL8* (homologous genes of *TaD53*) was markedly increased, whereas the expression of *AtSPL2* (homologous genes of *TaSPL3*) and *AtBRC1* (homologous genes of *TaTB1*) was markedly reduced in the internodes of transgenic *Arabidopsis*. Besides, TaSPL6B, TaSPL3 and TaD53 interacted with one another, as demonstrated by yeast two-hybrid and bimolecular fluorescence complementation assays. Therefore, we speculated that TaSPL6B brought together TaD53 and TaSPL3 and enhanced the inhibition effect of TaD53 on TaSPL3 through integrating light and strigolactone signaling pathways, followed by suppression of TaTB1, a key repressor of tillering.

**Conclusions:**

As a whole, our findings contribute to a better understanding of how SPL genes work in wheat and will be useful for further research into how TaSPL6B affects yield-related traits in wheat.

**Supplementary Information:**

The online version contains supplementary material available at 10.1186/s12870-024-05429-2.

## Background

SPL proteins are plant-specific transcription factors with a highly conserved SBP (Squamosa promoter binding protein) domain. This domain promotes the transcription of target genes by binding to motifs with the consensus sequence “TNCGTACAA” (in which “N” stands for any base) [1]. SPLs are first identified in *Antirrhinum majus* due to their capacity to bind the promoter of the SQUAMOSA gene [2]. Since the first identification of SPLs in *Antirrhinum majus*, SPLs have been found in the genomes of numerous plant species, including *arabidopsis thaliana* [3], maize [4], tomato [5], rice [6], *populus* [7], *gossypium hirsutum* [8], wheat [9, 10] and *paulownia fortune* [11]. SPLs have been shown to have many functional effects in various studies. For example, SPLs regulate many extensive networks such as vegetative-to-floral transition [12–15], shoot development [16], fruit development [5], grain quality and yield [17–19], shoot/panicle branching and male fertility [20, 21]. Interestingly, approximately two-thirds of SPLs contain the complementary sites of microRNA156 (miR156), which targets SPLs for cleavage and repress their translation and then regulate a large network of genes related to plant development and growth [22–26].

Previous studies have confirmed the critical role of SPLs in regulating branching and florescence and relevant regulatory mechanisms have been investigated in model plants. Strigolactone (SL) and light signaling pathways coordinately regulate branching involving a conserved D53-SPL-TB1/FC1/BRC1 pathway. SPLs can activate key inhibitory genes for tillering such as *TB1* in maize, *FC1* in rice and *BRC1* in *Arabidopsis*, which are orthologous class II TCP transcription factors [27]. SL are recently identified carotenoid-derived phytohormones that play a prominent role in repressing shoot branching [28]. Significant progress has been made in understanding the perception and signal propagation mechanisms of SL signaling in model plants. In rice, the ubiquitination and degradation of DWARF53 (D53) are triggered when SL signaling are perceived by a receptor complex made up of F-box leucine-rich repeat protein D3 and the hydrolase DWARF14 (D14). D53, a repressor of SL signaling, can form a repressor-corepressor-nucleosome complex with TOPLESS (TPL) and TOPLESS-related (TPR), thereby inhibiting the transcriptional activation activity of associated transcription factors such as SPLs [29, 30]. AtD14, the D3 ortholog MAX2 and the D53-like proteins SMXL6/7/8 make up the SL signaling receptor complex in *Arabidopsis*. SMXL6/7/8 can interact with TPR2 and function as transcriptional repressors [31–33]. Previous studies also reported that the phytochrome A (phyA)-mediated far-red light signaling components FHY3 and FAR1 can upregulate the expression of *SMXL*s in the light signaling pathway. The transcriptional activities of SPL9 and SPL15 are inhibited through direct interactions with SMXL6/7/8, followed by the suppression of BRC1, thus affecting tillering [34].

Exogenous cues (gibberellic acid (GA) signaling, age of a plant and sugar assimilates) and endogenous cues (light and temperature) have been hypothesized to control the timing of blooming in plants. When all factors are considered, there are five main pathways: photoperiod, age, vernalization, gibberellic acid and autonomous [35]. In photoperiod pathway, flowering is regulated through the response of plant to the day length and quality of light perceived. The age pathway is considered an endogenous pathway. The vernalization pathway involves exposing seeds to cold for a long period to accelerate flowering. The requirement for GA in regular flowering patterns is referred to as the GA pathway. Other endogenous regulators form the autonomous pathway. These pathways convey the signals through various floral integrators to build an integrated regulatory network to control blooming time [36, 37]. The SPL family control flowering time in the model plant *Arabidopsis* and overexpression of SPLs causes early flowering and abnormal inflorescence [38–40]. In *Arabidopsis*, the majority of SPL genes are targeted by miR156, miR157 and miR172, which positively and directly regulate downstream flowering genes [41–43]. This mechanism has also been demonstrated in other species [44, 45].

Wheat (*Triticum aestivum*), a major staple crop, is an excellent human source of carbohydrates and proteins [46]. Global demand for wheat is growing with the increasing population; however, wheat breeding faces significant challenges in maintaining stable and high yields. Therefore, mining valuable genes will facilitate the breeding of new varieties and increase wheat yield. This study aims to isolate TaSPL6B from wheat and evaluate its function, enriching our understanding of SPL proteins in wheat and contributing to further investigation of the effects of TaSPL6B on yield-related traits.

## Results

### Bioinformatics analysis of three TaSPL6 homologs

**Fig. 1:**
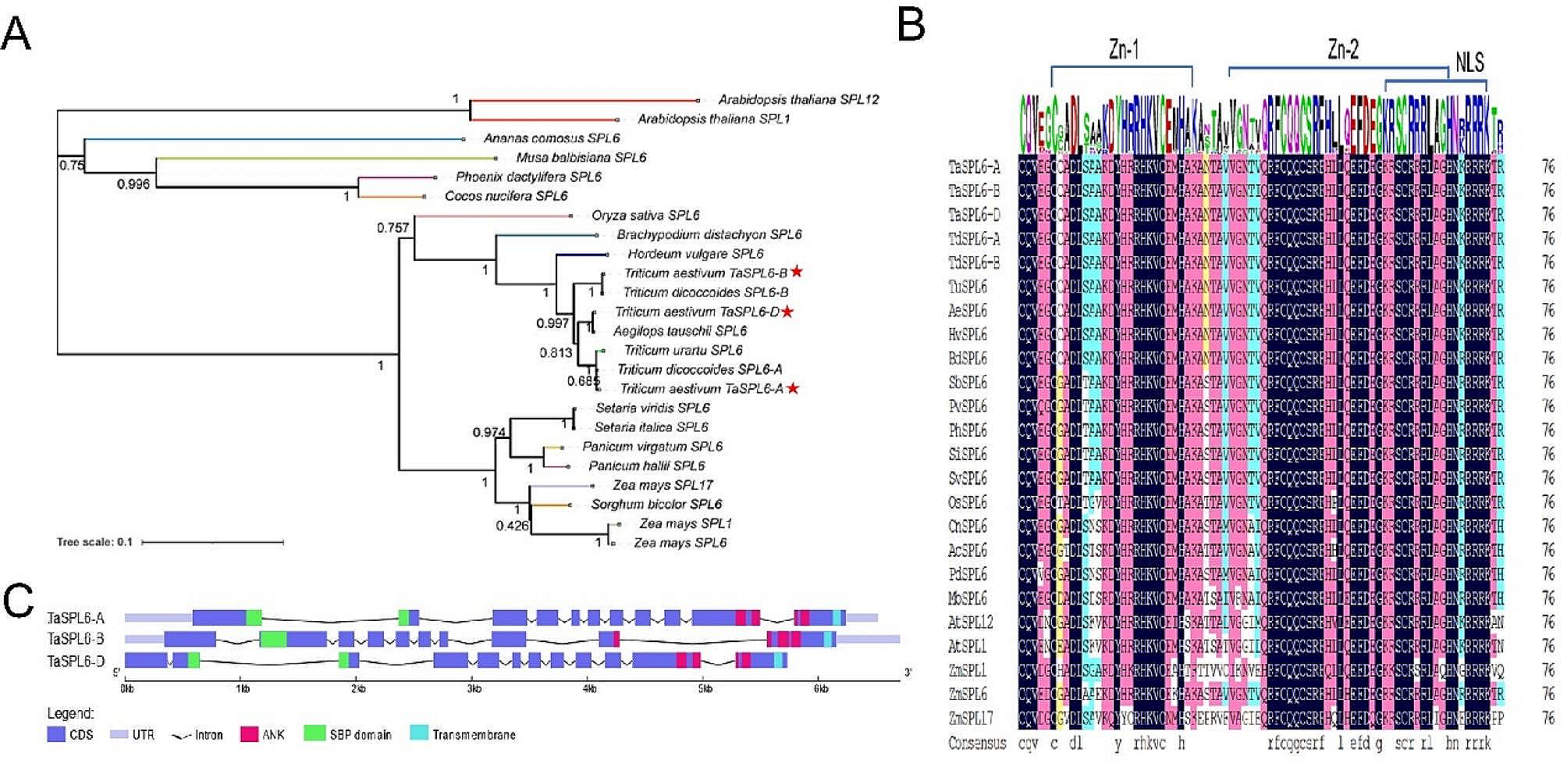
Phylogenetic analysis and multiple sequence alignment of conserved SBP domain based on the blast results and gene/protein structures prediction of the three homologs of TaSPL6 in Wheat. **A** The phylogenetic relationship between three homologs of TaSPL6 and other 21 blasted SPL proteins was shown in the neighbor-joining tree, which was constructed with 1000 bootstrap iterations. Three TaSPL6 homologous proteins were marked with red stars. **B** The multiple sequence alignment of conserved SBP domain based on 24 SPL proteins. Sequences LOGO view based on the result of sequence alignment was placed at the top of the figure. Zinc-binding sites and NLS structure were also labeled. Different colors represent the conservative degree of amino acids: black, 100% conserved; amaranth, 75–100% conserved; Light blue, 50–75% conserved; yellow, 33–50% conserved; white, less than 33% conserved. **C** Gene and protein structures analysis of the three homologs of TaSPL6. The parts of CDS, UTR, Intron, ANK, SBP domain and Transmembrane were represented in purple, lilac colour, black, rose red, green and light blue colors, respectively

To better understand TaSPL6 proteins, we performed bioinformatics analysis of three TaSPL6 homologs (Fig. 1). Based on BLAST results, the NJ phylogenetic tree with 24 SPL proteins was constructed (Fig. 1A). Detailed information on these proteins was provided in Table S1. The dendrogram showed that the SPL family members were further divided into three subgroups. Three TaSPL6 homologous proteins showed high homology with SPL6A and SPL6B of *Triticum dicoccoides*, SPL6 of *Aegilops tauschii* and SPL6 of *Triticum urartu*, indicating a close evolutionary relationship among wheat, *Triticum dicoccoides*, *Aegilops tauschii* and *Triticum urartu*. Multiple sequence alignment revealed that the SBP domain had two zinc-finger structures and approximately 76 amino acid residues. A nuclear localization signal (NLS) was presented at the C-terminus of the domain, which is characteristic of plant transcription factors and drags the SBP-box gene into the nucleus to regulate the transcriptional expression of its downstream genes (Fig. 1B). Structural analysis of SBP domain further demonstrated the evolutionary conservatism of SPL proteins. The 20 most statistically significant motifs, named motif 1–20, were selected to describe the motif pattern in 24 SPL proteins. The result of the motif analysis indicated that TaSPL6 homologous proteins have conserved motifs (Fig. S1). To evaluate the evolution of TaSPL6 homologous proteins, we further investigated the structural organization of their gene and protein domains. The results revealed that the structural organization of three proteins were similar, each member had a conserved SBP domain, ANK domain and transmembrane domain in the proteins and exons and introns in the genes, which further indicated TaSPL6 homologous proteins are conservative in evolution (Fig. 1C).

Considering the balanced expression pattern of the TaSPL6 gene triads in the A, B and D subgenomes of Azhurnaya (Fig. S2), we selected *TaSPL6B* for further functional studies. We cloned *TaSPL6B* which has a nucleotide sequence length of 2886 bp and encoding 961 amino acids. It has an aliphatic index of 79.17, pI of 5.54 and a molecular weight of 105 kDa. TaSPL6B is also a hydrophilic protein by its grand average hydropathicity of -0.294 (Table S2). NetPhos prediction demonstrated that the protein sequence contains numerous phosphorylation sites. Secondary structure analysis revealed that the protein comprised alpha helices, random coils, extended strands and beta turns, accounting for 35.69%, 47.55%, 12.17% and 4.58%, respectively. The protein possessed a C-terminal transmembrane domain, as predicted by TMHMM 2.0. (Fig. S3). Analysis of the *cis*-acting element showed that *TaSPL6B* contained regulatory elements that are responsible for light, GA, anaerobic induction, meristem/endosperm expression, zein metabolism regulation, low-temperature, abscisic acid and drought-inducibility responsiveness, suggesting that *TaSPL6B* plays important roles in multiple signaling pathways (Table S3).

### Ectopic expression of TaSPL6B in *Arabidopsis* results in early flowering

**Fig. 2:**
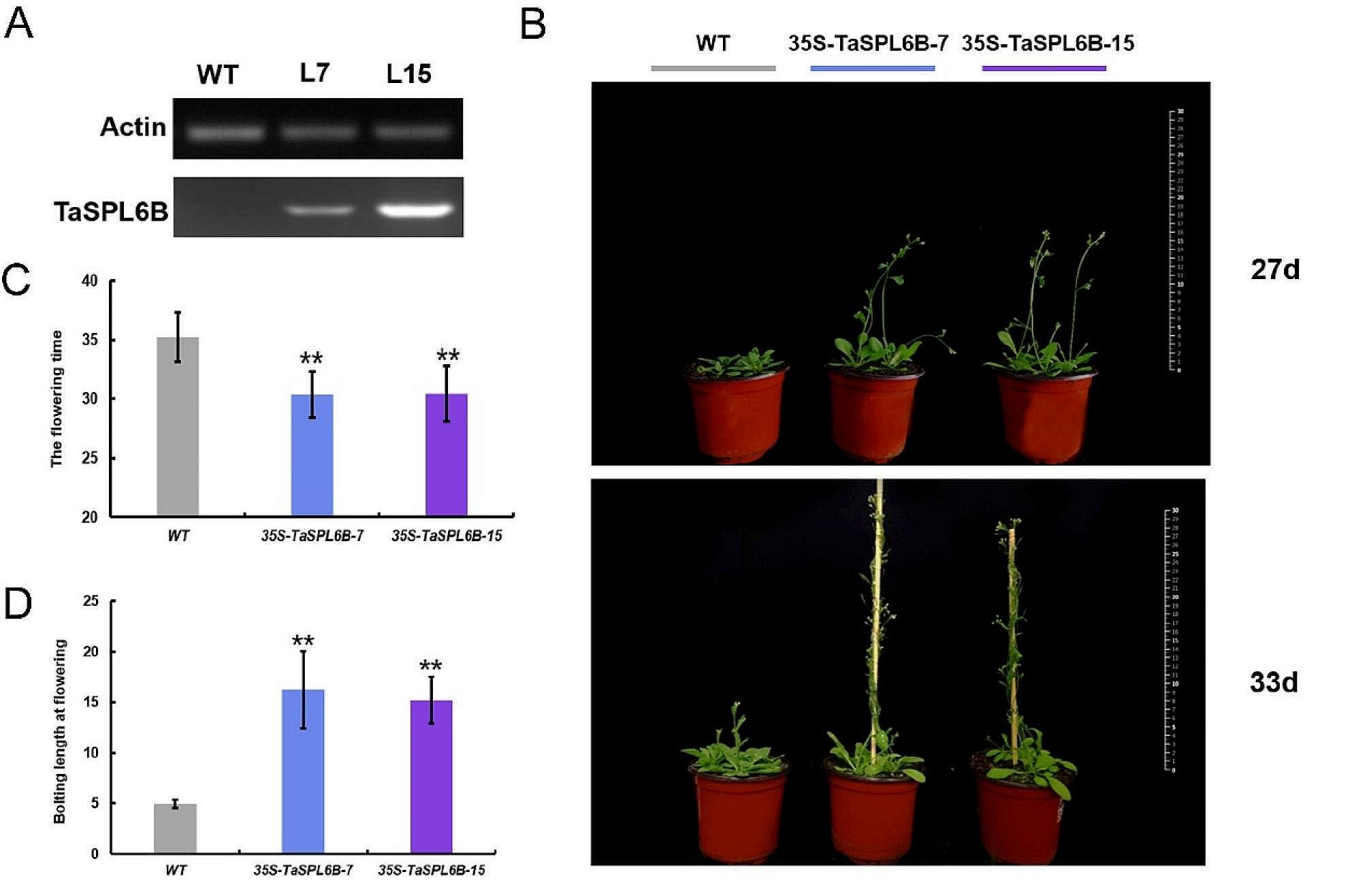
Overexpression of TaSPL6B causes early flowering. **A** TaSPL6B was highly expressed in two overexpressed lines based on semi-quantitative PCR. **B** Flowering phenotypes of wild-type (WT) plant and two overexpressed 35 S-*TaSPL6B* (OE) lines. **C** The statistics of flowering time. Overexpressing *TaSPL6B* in Arabidopsis produced early flowering than wild-type (WT) plant. **D** The statistics of bolting length at flowering. The bolting length of two overexpressed 35 S-TaSPL6B (OE) lines were longer than that of wild-type (WT) plant when flowering. The values are means ± SD (Student’s t-test, **, *P* < 0.01)

To investigate the phenotype and expression level, we selected two homozygous lines 35 S-*TaSPL6B*-7 and 35 S-*TaSPL6B*-15. Semi-quantitative PCR confirmed that TaSPL6B was highly expressed in the two lines (Fig. 2A). Phenotypic observations showed that *TaSPL6B*-OE lines flowered earlier than the wild-type (WT) plants; The *TaSPL6B*-OE lines flowered after 30 days on average, whereas WT flowered after 35 days on average (Fig. 2B, C). Furthermore, compared to WT plants, the bolting length of transgenic lines was significantly higher at the first flowering stage (Fig. 2B, D). The results demonstrated that ectopic expression of *TaSPL6B* in *Arabidopsis* results in early flowering and a notable difference in bolting length during the flowering period. Statistical data on phenotypes are also presented (Table S4).

### Ectopic expression of TaSPL6B in *Arabidopsis* promotes shoot branching

**Fig. 3:**
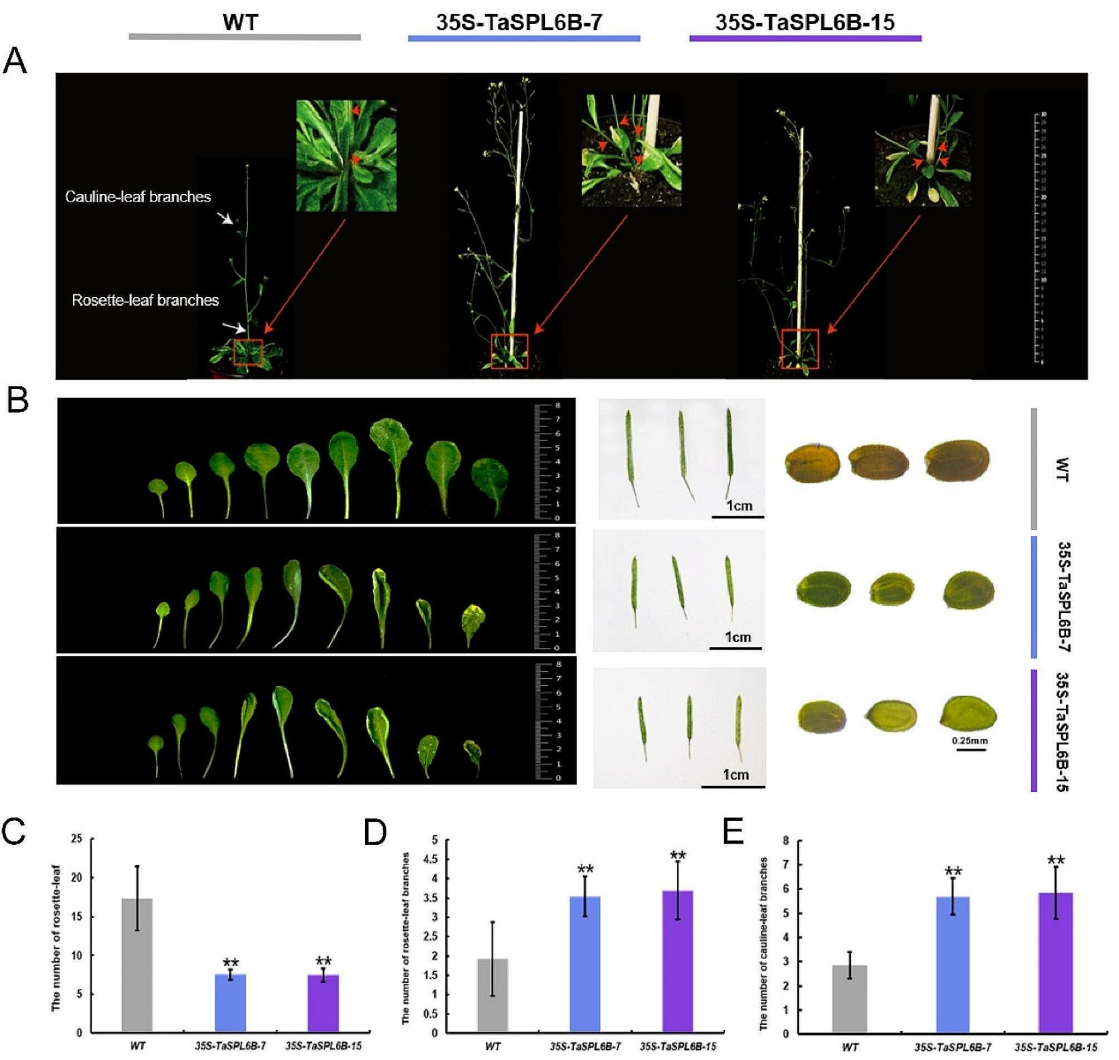
Overexpression of *TaSPL6B*causes excessive branching. **A** Branching phenotypes of wild-type (WT) plants and two overexpressed 35 S-*TaSPL6B* (OE) lines on 41 days-old. Overexpressing *TaSPL6B* in Arabidopsis produced more branches than wild-type (WT) plants. **B** Differences in rosette-leaves, siliques and seeds phenotypes of wild-type (WT) plants and two overexpressed 35 S-*TaSPL6B*f (OE) lines. **C** The number of rosette-leaf of wild-type (WT) plants and two overexpressed 35 S-*TaSPL6B* (OE) lines. **D** The number of rosette-leaf branches of wild-type (WT) plants and two overexpressed 35 S-*TaSPL6B* (OE) lines. **E** The number of cauline-leaf branches of wild-type (WT) plants and two overexpressed 35 S-*TaSPL6B* (OE) lines. The minimum length of the branches counted to obtain the total number is 1 centimeter (cm). The values are means ± SD (Student’s t-test, **, *P* < 0.01)

In 40-day-old plants, *TaSPL6B*-OE lines had a greater number of cauline-leaf and rosette-leaf branches compared to WT plants (Fig. 3A). Statistical analysis revealed that two independent transgenic lines had fewer and smaller rosette leaves at the early-bloom stage and their leaves were curled compare to WT plants. Besides, the transgenic lines had shorter silique lengths and smaller seed sizes, with seeds also having a distinct color compare to WT plants. At maturity, seeds from WT plants were brown, while those from *TaSPL6B-*OE plants were yellow (Fig. 3B, C). At least three rosette-leaf branches were observed in *TaSPL6B*-OE lines and almost all of the rosette-leaf buds developed into rosette-leaf branches (Fig. 3D). Similarly, the number of cauline-leaf branches was increased in *TaSPL6B*-OE lines (more than five) compared to WT (three) in 40-day-old plants (Fig. 3E). The findings underscore the diverse roles of TaSPL6B as a transcription factor, crucially promoting tillering in transgenic *Arabidopsis*.

### Expression pattern analysis of branching-related genes

**Fig. 4:**
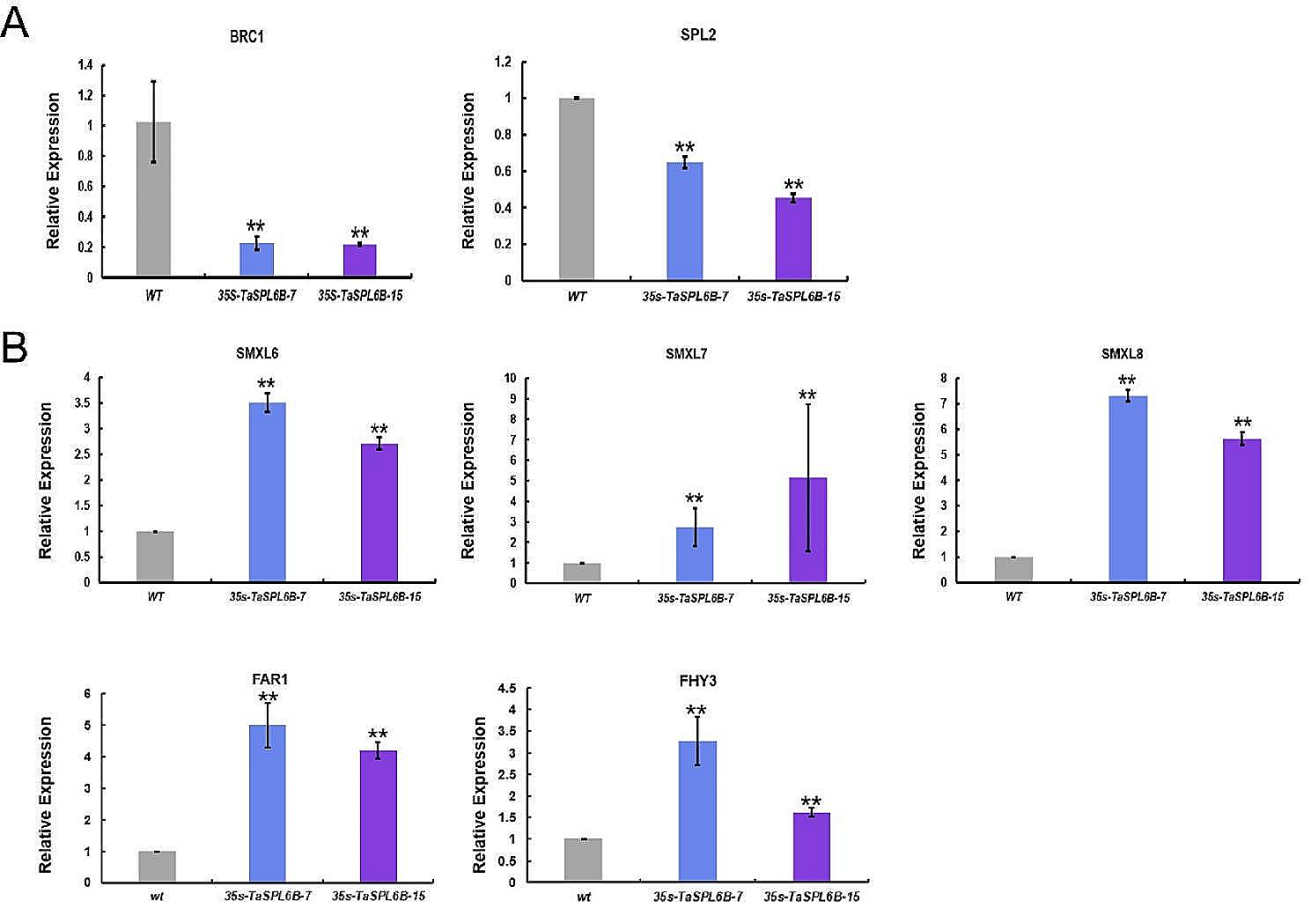
Expression patterns analysis of branch-related genes. **A** Expression patterns analysis of *AtBRC1* and *AtSPL2* in wild type (WT) and two 35 S-*TaSPL6B* (OE) lines. **B** Expression patterns analysis of *AtSMXL6*, *AtSMXL7*, *AtSMXL8*, *AtFHY3* and *AtFAR1* in wild type (WT) and two overexpressed 35 S-*TaSPL6B* (OE) lines. The values are means ± SD (Student’s t-test, **, *P* < 0.01)

We detected the expression of branching-related genes in the internodes of WT and *TaSPL6B*-OE lines for functional exploration, including *AtBRC1* (homolog of *TaTB1*, local branch development regulatory gene), *AtSPL2* (homolog of *TaSPL3*) and *AtSMXL6*/*AtSMXL7*/*AtSMXL8* (homologs of *TaD53*). In *Arabidopsis*, the expression of *AtSMXL6* and *AtSMXL7* can also be enhanced by *AtFHY3* and *AtFAR1* in light signaling pathway [34], we further detected the expression of *AtFHY3* and *AtFAR1*. The results showed that the expression levels of *AtSPL2* and *AtBRC1* were markedly reduced (Fig. 4A), while *AtFHY3/FAR1* and *AtSMXL6/AtSMXL7/AtSMXL8* were markedly increased in *TaSPL6B*-OEs compared with WT (Fig. 4B). Due to *AtSMXL6/AtSMXL7/AtSMXL8* are key members of SL signaling and *AtSMXL6* and *AtSMXL7* can also be enhanced by *AtFHY3* and *AtFAR1* in light signaling pathway, which further indicated light and SL signaling pathways play an important role in regulating tillering. All the qPCR data are also shown (Table S5).

### Transcriptional activation activity analysis and subcellular localization of TaSPL6B protein

**Fig. 5:**
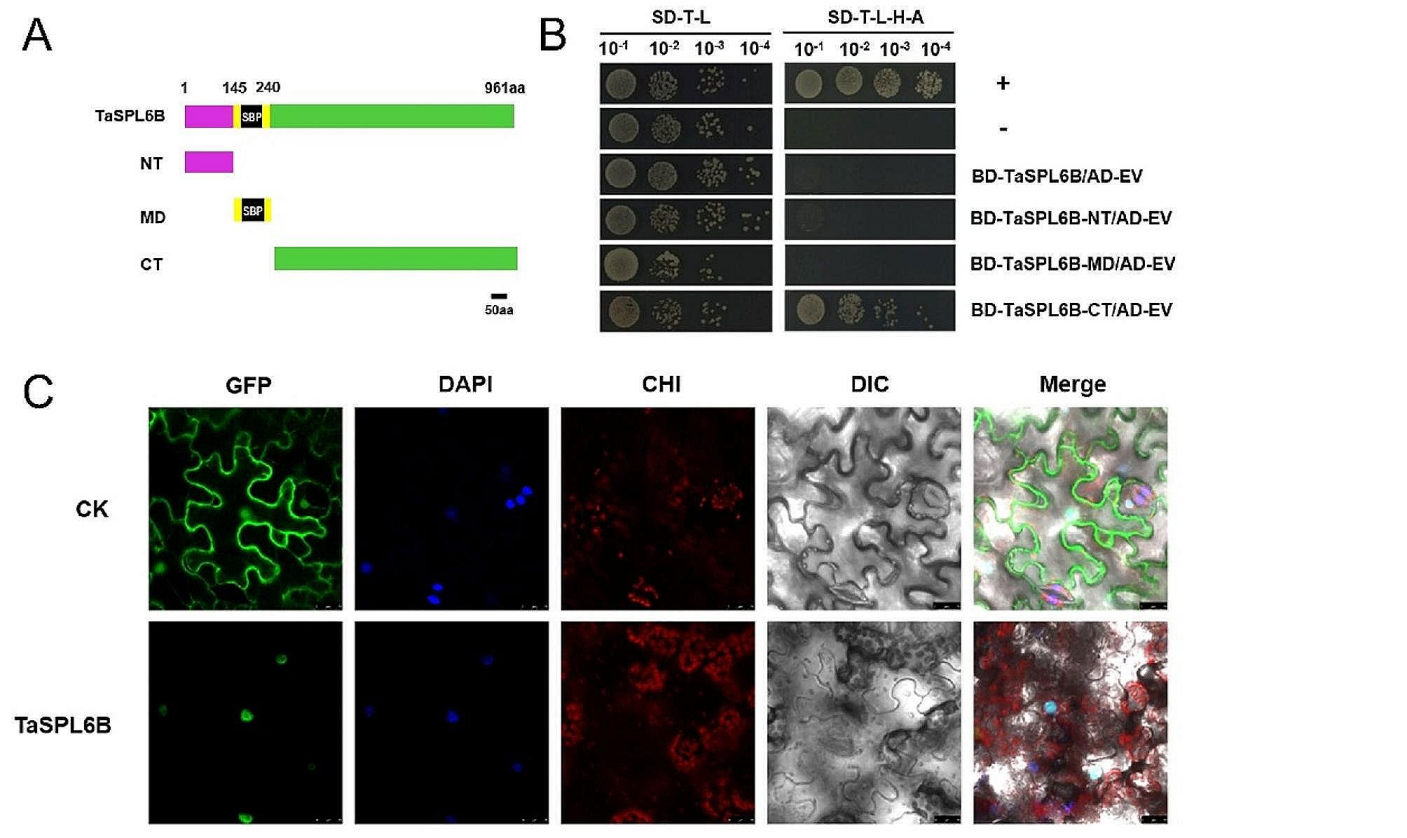
Analysis of the transcriptional activation activity and subcellular localization of TaSPL6B protein. **A** Schematic diagram of each fragment of TaSPL6B. **B** Transcriptional activation activity test of TaSPL6B and each fragment of TaSPL6B. The SD-T-L refers to media lacking tryptophan and leucine, and SD-T-L-H-A refers to media lacking tryptophan, leucine, histidine, adenine. AD: pGADT7; BD: pGBKT7. **C** Subcellular localization of the TaSPL6B-GFP protein in tobacco leaf epidermal cells

Self-activation assay revealed that the yeast cells containing BD-*TaSPL6B* and AD-EV could grow on SD-Trp-Leu but could not on SD-Trp-Ade-His-Leu, indicating that the full-length of TaSPL6B had no self-activation effect in transgenic yeast cells (Fig. 5). We divided TaSPL6B protein sequence into three parts: N-terminus (NT, 1-145 aa), middle part (MD, 146–240 aa) and C-terminus (CT, 241–961 aa) (Fig. 5A). Subsequently, we observed that the autoactivation effect only appeared in the CT, while the MD and NT of TaSPL6B might consist of transcriptional suppression domains that suppress the self-activation effect of TaSPL6B (Fig. 5B). In subcellular localization experiment, *N. benthamiana* leaf cells containing pCAMBIA2300-TaSPL6B-GFP had a high green fluorescence signal in the nucleus, whereas free GFP protein had a high green fluorescence signal in the plasma membrane and nucleus (Fig. 5C), suggesting a typical transcription factor role for TaSPL6B.

### TaSPL6B interacts with TaD53 and TaSPL3 in vitro and in vivo

**Fig. 6:**
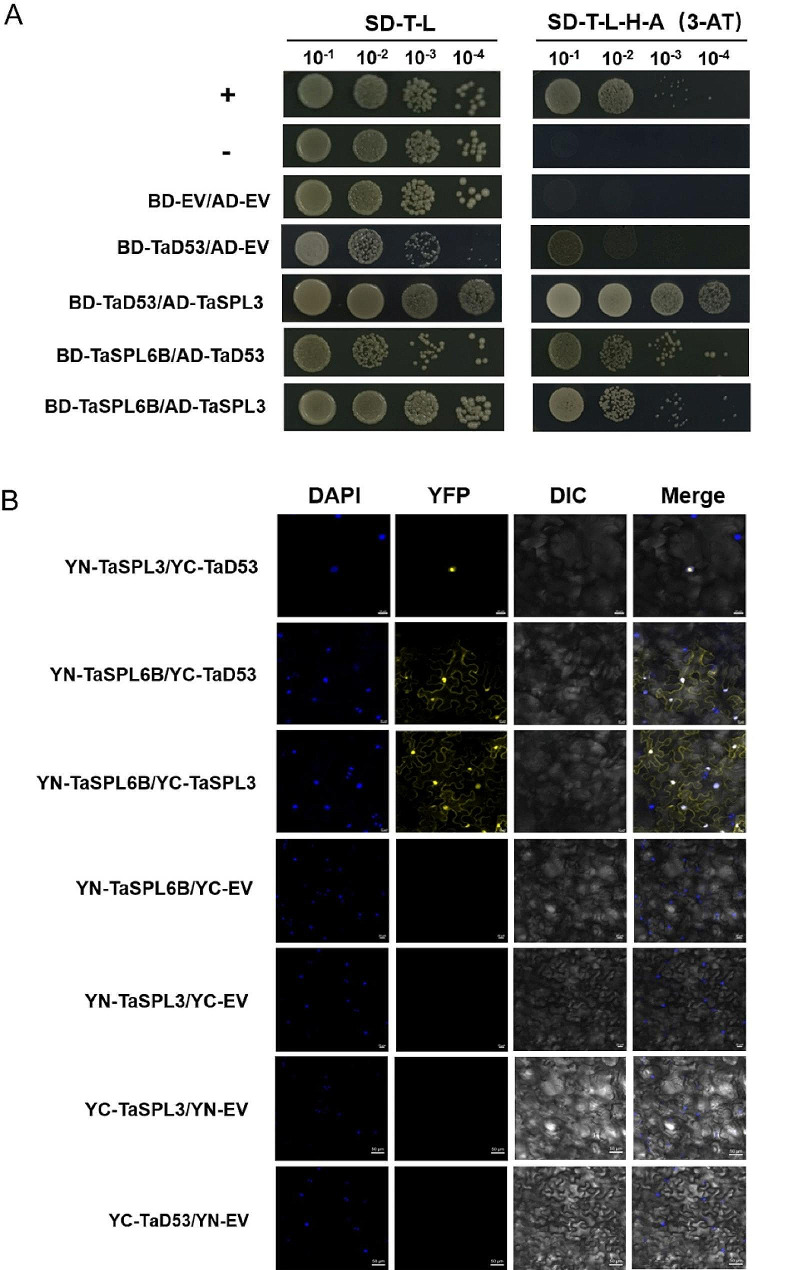
TaD53 TaSPL3 and TaSPL6B interact with one another. **A** Yeast two-hybrid results of TaD53, TaSPL3 and TaSPL6B proteins. The SD-T-L refers to media lacking tryptophan and leucine, and SD-T-L-H-A(3-AT) refers to media with 3-AT lacking tryptophan, leucine, histidine, adenine. AD: pGADT7; BD: pGBKT7. **B** BiFC assays results of TaD53, TaSPL3 and TaSPL6B proteins. The TaSPL6B coding regions were cloned into the pEarleygate201-YN (nYFP) vector. The TaD53 coding regions were cloned into pEarleygate202-YC(cYFP) vector and TaSPL3 coding regions were cloned into both pEarleygate201-YN (nYFP) vector and pEarleygate202-YC(cYFP) vector

The interactions between TaSPL6B and branching-related proteins were examined to investigate the molecular mechanism through which TaSPL6B regulates shoot branching (Fig. 6). A previous study demonstrated that TaD53 may interact with TaSPLs and regulate tillering in wheat [47]. Therefore, we investigate the interaction between TaD53 and TaSPL6B and established interactions between and TaD53 and TaSPL3 as a positive control. As the TaD53 protein has self-activation activity, 3-AT was added to the yeast two-hybrid assay culture medium to prevent erroneous positive results. In addition, a lot of transcription factors can physically interact with other members of the same family to form homodimers; therefore, we also explored the interaction between TaSPL3 and TaSPL6B [48]. Yeast two-hybrid assay confirmed that the yeast cells containing BD-*TaSPL6B*/AD-*TaD53*, BD-*TaSPL6B*/AD-*TaSPL3* and BD-*TaD53*/AD-*TaSPL3* could grow on SD-Trp-Ade-His-Leu (3-AT) plate, indicating potential interactions between these proteins in vitro of plant (Fig. 6A). BiFC assays were performed to study the interaction of these proteins in vivo of plant and the results revealed that TaSPL6B interacted with TaSPL3 and TaD53 in the nucleus and cell membrane, whereas TaSPL3 interacted with TaD53 in the nucleus (Fig. 6B).

## Discussion

### *TaSPL6B* exhibits diverse functions in transgenic *Arabidopsis*

According to previous research, *TaSPLs* regulated growth and development of wheat. In rice, for instance, ectopic expression of *TaSPL20* and *TaSPL21* both promoted panicle branching [49]. Through the SL signaling pathway, *TaSPL3/17* regulated plant architecture by targeting and activating *TaTB1* and *TaBA1* [47]. Through auxin and brassinosteroid signaling, *TaSPL8* was able to change the angle of the leaf [50]. *TaSPL13* could regulate inflorescence architecture and development when it was overexpressed in rice or wheat [51]. Mutations in *TaSPL13*-B and *TaSPL13*-AB displayed a pleiotropic effect, leading to an increased number of seeds per spike, decreased tiller number, increased grain width, reduced height and early flowering [26]. The plant height, panicle length, spikelet count and thousand-grain weight of *TaSPL14* knock-out plants were all reduced and it might be able to regulate the growth of wheat spikes through the ethylene-response pathway [52]. *TaSPL7A/B/D* and *TaSPL15A/B/D* might regulate plant height, tillering and panicle size. The hexa-mutants *TaSPL7*-aabbdd and *TaSPL15*-aabbdd increased the tiller number, decreased spike length and reduced spikelet number after being knocked out using the CRISPR/cas9 method [53]. Here, we identified *TaSPL6B* gene in wheat and characterized its function in *Arabidopsis*. We found that the *TaSPL6B*-overexpressing plants exhibited increased branching and had distinct seeds color compared with WT. Moreover, transgenic plants showed earlier flowering, fewer and smaller rosette leaves, shorter silique lengths and smaller seed sizes than WT (Figs. 2 and 3). All results suggested that *TaSPL6B* has diverse functions as transcription factor and could be relevant for wheat breeding.

### TaSPL6B integrates light and SL signaling pathways to regulate tillering

**Fig. 7:**
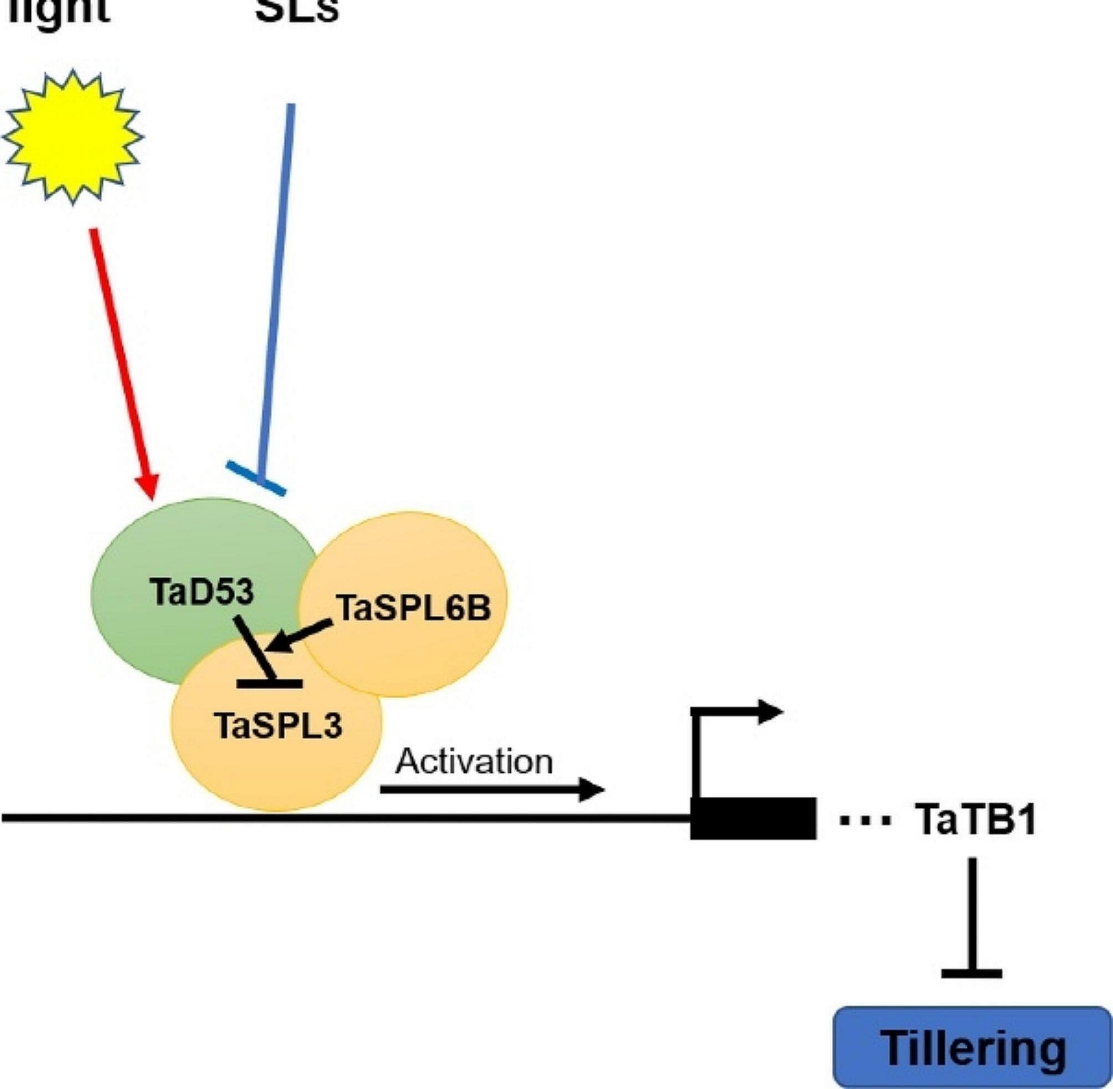
A Simplified schematic model depicting TaD53, TaSPL3 and TaSPL6B interact with one another and integrate Light and Strigolactone signal pathways to regulate tillering. TaD53, TaSPL3 and TaSPL6B interact with one another in Light and SL signal pathway, which suppress the transcriptional activation activity of TaSPL3, leading to suppress transcription of TaTB1, a key repressor of branching, thus promoting branching. Red line: Light signal pathway; Blue line: SL signal pathway; Arrow: activate; Bar: repress

Among many traits, tillering is important for crop architecture and determines grain yield. In rice [54], maize [21], *Arabidopsis* [55] and wheat [47], the conserved D53-SPL-TB1/FC1/BRC1 signaling pathway had been found to be the mechanism by which D53 controls tillering. D53 could physically interact with SPL transcription factors to block their ability to activate *TB1* transcription, thereby enhancing tillering. In *Arabidopsis*, the expression of *TaD53* homologous genes *SMXL6* and *SMXL7* could also be enhanced by *FHY3* and *FAR1* in light signaling pathway [34]. The *TaSPL6B*-OEs in this study showed enhanced expression of homologous genes of *TaD53* (*AtSMXL6/AtSMXL7/AtSMXL8*) and *AtFHY3/FAR1*, whereas homologous genes of *TaSPL3* (*AtSPL2*) and *TaTB1* (*AtBRC1*) showed reduced expression, indicating that *TaSPL6B* integrates light and SL signaling pathways to regulate tillering. Yeast two-hybrid and BiFC assays further confirmed that TaSPL6B, TaD53 and TaSPL3 interact with one another in *vitro* and in *vivo*. Based on all these findings in transgenic *Arabidopsis*, there is a putative model in wheat when Ta*SPL6B* overexpression, *TaD53* expression shows an overall increasing trend through the light and SL signaling pathways. Furthermore, TaD53 may block the transcriptional activation activities of TaSPL3 by interacting with it, while TaSPL6B may contribute to bringing TaD53 and TaSPL3 together to form a trimer. TaSPL3 functions to enhance the expression of the downstream gene *TaTB1*, so its inhibition will lead to the suppression of *TaTB1*, a key repressor of branching, thereby promoting tillering (Fig. 7).

### The mechanism by which TaSPL6B regulates flowering through photoperiod and GA pathways needs to be further explored in TaSPL6B

The gene showed high expression in the stage of anthesis-stigma-ovary during the entire Azhurnaya growth period in Fig. S4, indicating that *TaSPL6B* plays an important role in flowering. The mechanism by which SPLs regulate flowering had been the subject of numerous studies. For example, *AtSPL9* could enhance the expression of *AP1*, *FUL* and *SOC1* genes to promote flowering. Besides, *AtSPL9*, together with LFY, recruited DELLA proteins to the regulatory region of the *AP1* gene in the GA pathway [56, 57]. The major age and GA pathways converged in regulating of *AtSPL15* at both post-transcriptional and post-translational levels to promote flowering [58]. *AtSPL3*, *AtSPL4* and *AtSPL5* also played significant roles in the GA pathway, binding to the promoters of *AP1*, *LFY* and *FUL* to initiate flowering. Moreover, the interaction of AtSPL3/AtSPL4/AtSPL5 with FD made miRNA-mediated developmental aging and FT-mediated photoperiodic signals integrated into the flowering pathways [59]. Above studies suggested that the photoperiod and GA pathways are crucial in flowering regulation by SPLs. In this study, the *TaSPL6B*-OE lines began to bloom on average of 30 days, which was 5 days earlier than WT plants (Fig. 2B, C). Analysis of *cis*-acting regulatory elements suggested that *TaSPL6B* may regulate flowering through the photoperiod and GA pathways. In the upstream of *TaSPL6B* sequence, *cis*-acting regulatory elements involved in light and GA responsiveness were found and contained G-box, TCCC-motif and P-box (Table S3). Further investigation on how *TaSPL6B* regulates flowering in *Arabidopsis* would be valuable.

## Conclusions

Three TaSPL6 homologous proteins were subjected to a comprehensive bioinformatics analysis. Ectopic expression of *TaSPL6B* in *Arabidopsis* increased the number of branches and caused early flowering. Subcellular localization analysis showed TaSPL6B was localized in the nucleus. The full-length sequence of TaSPL6B had no transcriptional activity. Further exploration of expression patterns revealed that *TaSPL6B* was highly transcribed in internodes of *TaSPL6B*-OEs. Additionally, the homologous genes of *TaD53* (*AtSMXL6/AtSMXL7/AtSMXL8*) were markedly increased while the homologous genes of *TaSPL3* (*AtSPL2*) and *TaTB1* (*AtBRC1*) were markedly reduced in the internodes of *TaSPL6B-*OEs. Yeast two-hybrid and BiFC assays showed that TaSPL6B, TaSPL3 and TaD53 interact with one another. Therefore, we speculated that TaSPL6B might regulate branching by integrating the light and SL signaling pathways. Our study revealed that TaSPL6B has diverse functions in transgenic *Arabidopsis*, with potential implications for future wheat breeding.

## Methods

### Plant materials and growth conditions

All of the *Arabidopsis* stocks used in the study were created using a genetic background from Columbia (Col). After stratification for three days at 4 °C in the dark, *Arabidopsis* seeds were transferred to an incubator with long-day conditions at 22 °C and approximately 70% relative humidity. N. benthamiana was grown in an incubator at a constant temperature of 25 °C and a relative humidity of about 80%.

### Bioinformatics analysis

The protein sequences of three homologs of TaSPL6 were downloaded from the WheatOmics1.0 database (http://202.194.139.32/#), namely TaSPL6A (TraesCS4A03G0888200.1), TaSPL6B (TraesCS5B03G1243200.1) and TaSPL6D (TraesCS5D03G1120600.1). After that, the protein sequences were blasted against the NCBI (https://blast.ncbi.nlm.nih.gov/Blast.cgi). A neighbor-joining (NJ) phylogenetic tree was built based on the BLAST results using the MEGA7.0 software [60]. The SMART (http://smart.embl-heidelberg.de/smart) was used to confirm the domains of proteins. GSDS 2.0 (http://gsds.cbi.pku.edu.cn/index.php) was used to display domain architecture and exon-intron structure [61]. DNAMAN 6.0 was used to perform multiple sequence alignments of the SBP domain. Based on the alignment results, a sequence logo was generated by Weblogo (http://weblogo.berkeley.edu/logo.cgi). Conserved SPL motifs were identified using MEME (http://meme-suite.org/) and the results were shown using TBtools (https://github.com/CJ-Chen/TBtools/) [62]. The triad expression analysis was carried out as outlined previously [63]. The transcripts per million (TPM) values of the *TaSPL6* gene triad expression profile data for the entire Azhurnaya growth period were shown in Table S6 and were downloaded from www.wheat-expression.com. The R package GGTERN was used to create triangle plots [64]. ExPASy (https://web.expasy.org/protparam/) was used to analyze the physicochemical properties, molecular weight, instability index, isoelectric point (pI) and aliphatic index. ProtScale (https://web.expasy.org/protscale/) was used to examine the hydrophilicity. The NetPhos 3.1 server (http://www.cbs.dtu.dk/services/NetPhos/) was used for the analysis of the phosphorylation sites. The secondary structure was analyzed using NPS-SOPMA (https://npsa-prabi.ibcp.fr/cgi-bin/secpred_sopma.pl). The transmembrane area was analyzed using TMHMM 2.0 (

### Gene cloning, vector construction and subcellular localization of TaSPL6B

*TaSPL6B* was cloned and ligated into the SacI/XbaI-digested pCAMBIA2300-GFP vector. Agrobacterium tumefaciens (GV3101) was transformed into the recombinant vector pCAMBIA2300-*TaSPL6B*-GFP and the control vector pCAMBIA2300-GFP, respectively. The transformed GV3101 bacteria were cultured in yeast mannitol broth (YMB) supplemented with rifampicin (50 mg/L), gentamicin (50 mg/L) and kanamycin (50 mg/L) at 28 °C. After adjusting the OD_600_ of the transformed GV3101 bacteria in the infiltration buffer to 0.6, the leaves of 4-week-old N. benthamiana were infiltrated with the suspension cultures. The infiltrated leaves were examined using a confocal laser scanning microscope two days after the injection.

### Construction of transgenic plants

The floral dip method was used to transform the overexpression constructs pCAMBIA2300-*TaSPL6B*-GFP into the *Arabidopsis* ecotype Col-0 in order to generate *TaSPL6B* overexpressing (OE) lines [65]. To identify transgenic lines, T1 seeds were sown on 1/2 MS medium containing 50 mg/L kanamycin. The independent lines bearing a single copy of the transgene were determined by analysis of T_2_ seeds and confirmed as transgenic using PCR by amplifying 35 S:*TaSPL6B* fragment and bar gene. Homozygous lines from the T3 generation were used in all experiments and wild-type (Col-0) plants served as the control.

### Measurements of phenotype

The flowering time was measured when the flowers bloom. The bolting length at flowering was measured using the ruler. The number of rosette-leaf and cauline-leaf branches were measured on 41-days-old. The minimum length of the branches counted to obtain the total number was 1 cm. The phenotype of rosette-leaves were photographed every 3 days during the growth period and the siliques were photographed at 61-days-old. The seeds phenotypes were photographed using stereoscopic microscope.

### Quantitative RT-PCR analysis

After 50 days, total RNA was extracted from transgenic *Arabidopsis* tissue samples (rosette leaves, stem leaves, roots, stems and flowers) based on the manufacturer’s instructions. Using the Reverse Transcription System and 2 µg total RNA for reverse transcription. 2×SYBR Green qPCR Master Mix was used for qRT-PCR assay and expression levels of target genes were analyzed using the 2^–ΔΔCT^ method. Three replicates were used for the experiments.

### Transactivation activity analysis in yeast

The CDSs of *TaSPL6B* and three fragments were cloned into the pGBKT7 vector after being amplified using the gene-specific primers. Then the recombinant vectors (pGBKT7-*TaSPL6B*/pGADT7-EV, pGBKT7-*TaSPL6B*-CT/pGADT7-EV, pGBKT7-*TaSPL6B*-MD/pGADT7-EV and pGBKT7-*TaSPL6B*-NT/pGADT7-EV), positive control vector (pGBKT7-*53*/pGADT7-*T*) and negative control vector (pGBKT7-*Lam*/pGADT7-*T*) were transformed into AH109. The transformed strains were further cultured on SD-T-L (media lacking tryptophan and leucine) and SD-T-L-H-A (media lacking tryptophan, leucine, histidine and adenine) for 3–5 days at 30 °C.

### Yeast two-hybrid assay

The CDSs of *TaSPL6B*, *TaD53* and *TaSPL3* from wheat were inserted into the pGADT7 or pGBKT7 vector. The yeast strain AH109 was then co-transformed into the bait structure and each prey. SD-T-L and SD-T-L-H-A (3-AT) (media with 3-AT lacking tryptophan, leucine, histidine and adenine) media were used for culturing transformed yeast cells. On SD-Trp-Leu plates, yeast cells were chosen after three days at 30 °C and on the SD-Trp-Leu-His-Ade (3-AT) plates, further grown colonies with positive protein-protein interactions were selected.

### Bimolecular fluorescence complementation (BiFC) assay

The constructs used for BiFC assays were generated by an LR reaction between the vector pQBV3 and gateway vectors pEarleygate201-YN (nYFP) or pEarleygate202-YC (cYFP). Through infiltration, the GV3101 cultures containing constructs that expressed nYFP and cYFP fusion proteins were introduced into four-week-old N. benthamiana leaves in a 1:1 ratio. The plants were grown for 12 h in the dark, and then for 48 h in normal conditions in a growth chamber. The confocal microscope was used to image the YFP signal with an excitation laser set to 514 nm. Here is a list of all the primer sequences used (Table S7).

### Electronic supplementary material

Below is the link to the electronic supplementary material.

Supplementary Material 1

Supplementary Material 2

Supplementary Material 3

Supplementary Material 4

Supplementary Material 5

Supplementary Material 6

Supplementary Material 7

Supplementary Material 8

Supplementary Material 9

## Data Availability

The datasets analysed during the current study are available in WheatOmics1.0 database (http://202.194.139.32/#).
