# Supplementary material for: TaSPL6B, a member of the Squamosa promoter binding protein-like family, regulates shoot branching and florescence in Arabidopsis thaliana
Source: BMC Plant Biol. 2024 Jul 25;24:708. doi: 10.1186/s12870-024-05429-2 (PMC11271066; doi:10.1186/s12870-024-05429-2)

**Supplementary Fig. S1**. Motif composition models of 24 SPL proteins. Different motifs are color-coded according to the legend.


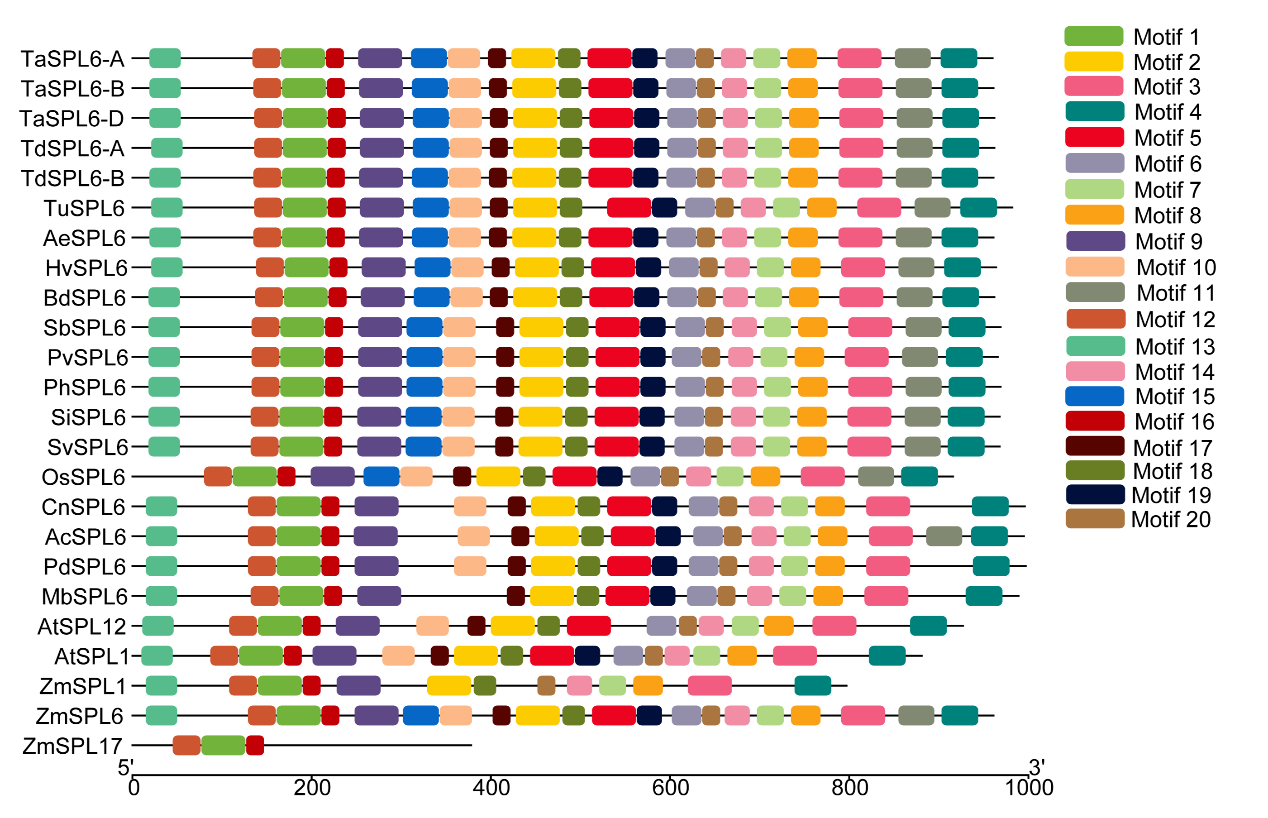


**Supplementary Fig. S2**. Expression balance for TaSPL6 gene triads from hexaploid wheat in different tissues was plotted in a triangular plot with the coordinates of each circle representing the normalized expression of A, B and D homoeologs. Triads are indicated by circles, with areas separated by blue lines indicating expression patterns that are balanced, dominant for one sub-genome homoeolog, or suppressed for one homoeolog, as previously described (Ramírez-González et al., 2018). Circle colours represent different tissues.


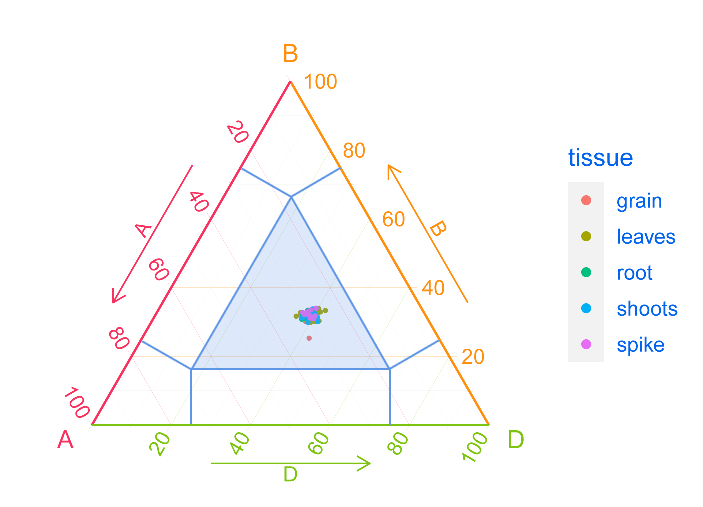


**Supplementary Fig. S3**. Bioinformatics analysis of TaSPL6B. **A** Hydrophilic and hydrophobic amino acid analysis of TaSPL6B. **B** Phosphorylation sites analysis of TaSPL6B. **C** Secondary structure prediction of TaSPL6B. Blue: Alpha helix; Orange: Random coil; Red: Extended strand; Green: beta turn. **D** Transmembrane domain analysis of TaSPL6B.


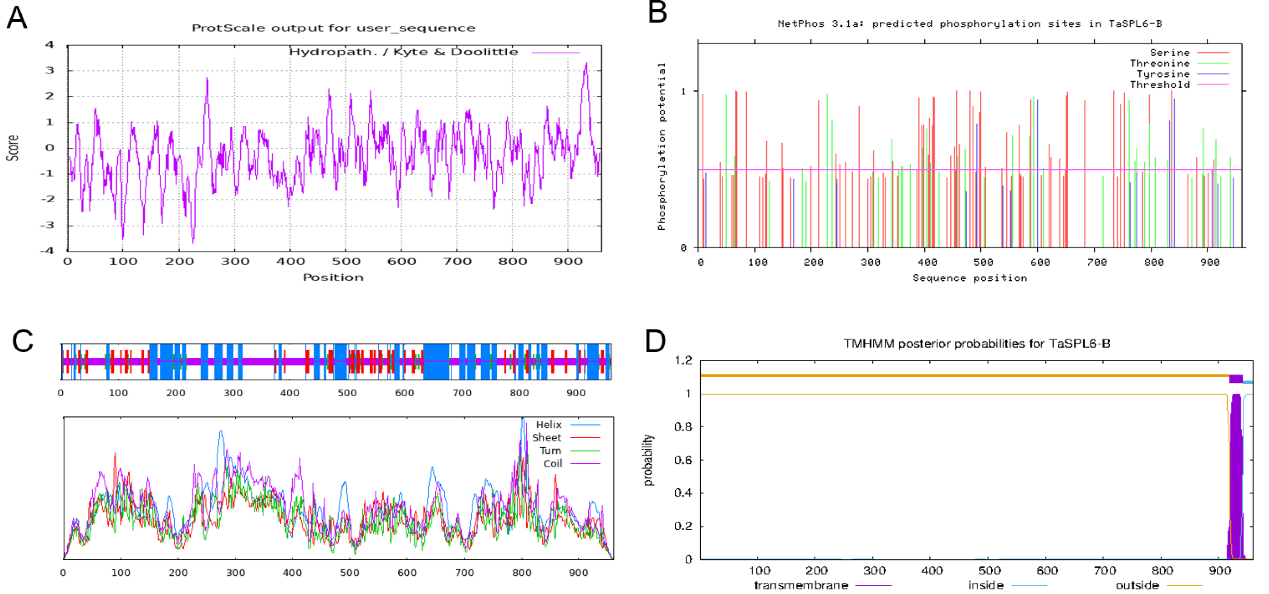


**Supplementary Fig. S4**. *TaSPL6* gene triad expression profile data for the entire Azhurnaya growth period.


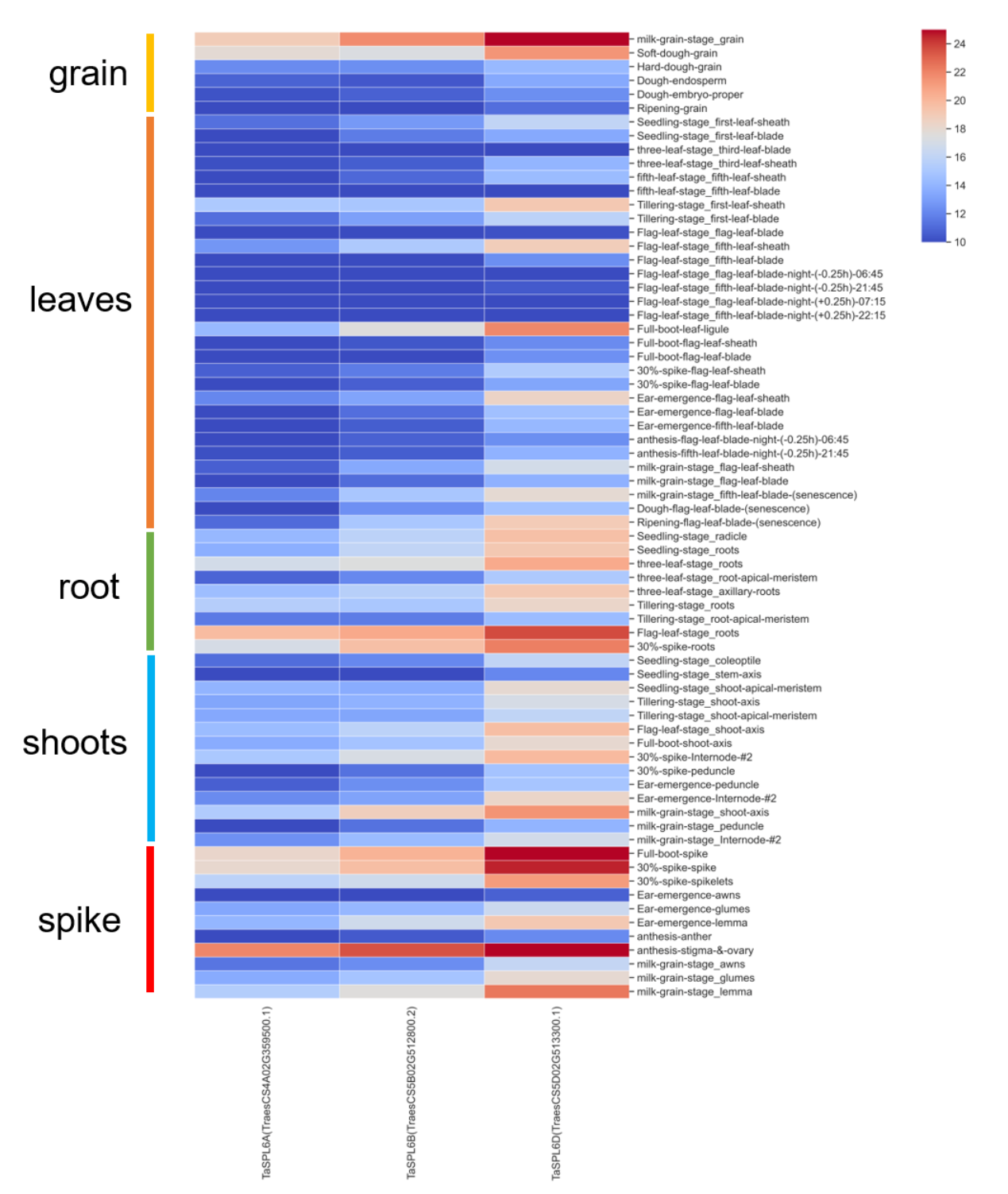

Supplement: Supplementary file 1 — Supplementary Material 1 [file 12870_2024_5429_MOESM1_ESM.docx]
