# Supplementary material for: TaSPL6B, a member of the Squamosa promoter binding protein-like family, regulates shoot branching and florescence in Arabidopsis thaliana
Source: BMC Plant Biol. 2024 Jul 25;24:708. doi: 10.1186/s12870-024-05429-2 (PMC11271066; doi:10.1186/s12870-024-05429-2)
Supplement: Supplementary file 2 — Supplementary Material 2 [file 12870_2024_5429_MOESM2_ESM.docx]

We have exhibited the original gel figure of Actin. As reference gene, Atactin can be expressed in WT and two overexpressed lines.

The semi-quantitative PCR result of Atactin


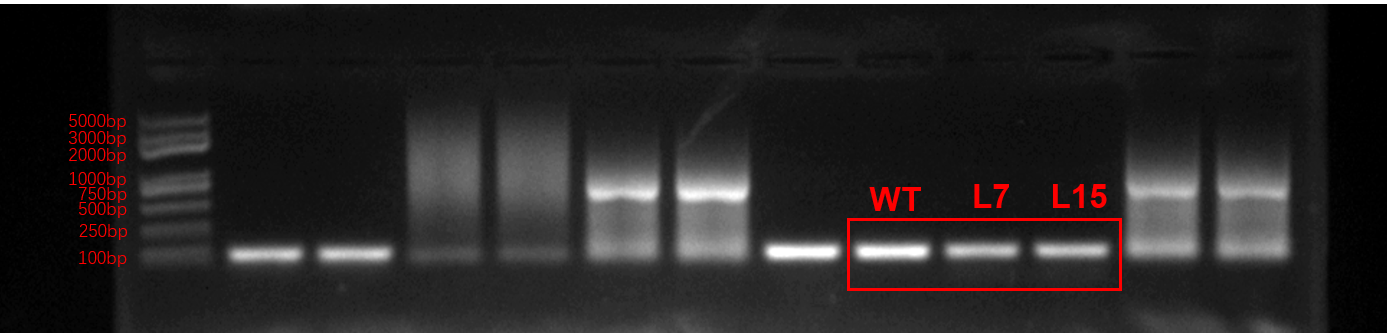


| q-Atactin-F: AGAAACCCTCGTAGATTGGCAC  q-Atactin-R: ACTCTCCCGCTATGTATGTCGC  Reference：Diao Y, Zhan J, Zhao Y, et al. GhTIE1 regulates branching through modulating the  transcriptional activity of TCPs in cotton and Arabidopsis[J]. Frontiers in Plant Science, 2019,  10: 1348. |
| --- |
| We have replaced the original gel figure of TaSPL6B with high-contrast and the latest results are  presented. TaSPL6B was highly expressed in two overexpressed lines. |

The semi-quantitative PCR result of TaSPL6B


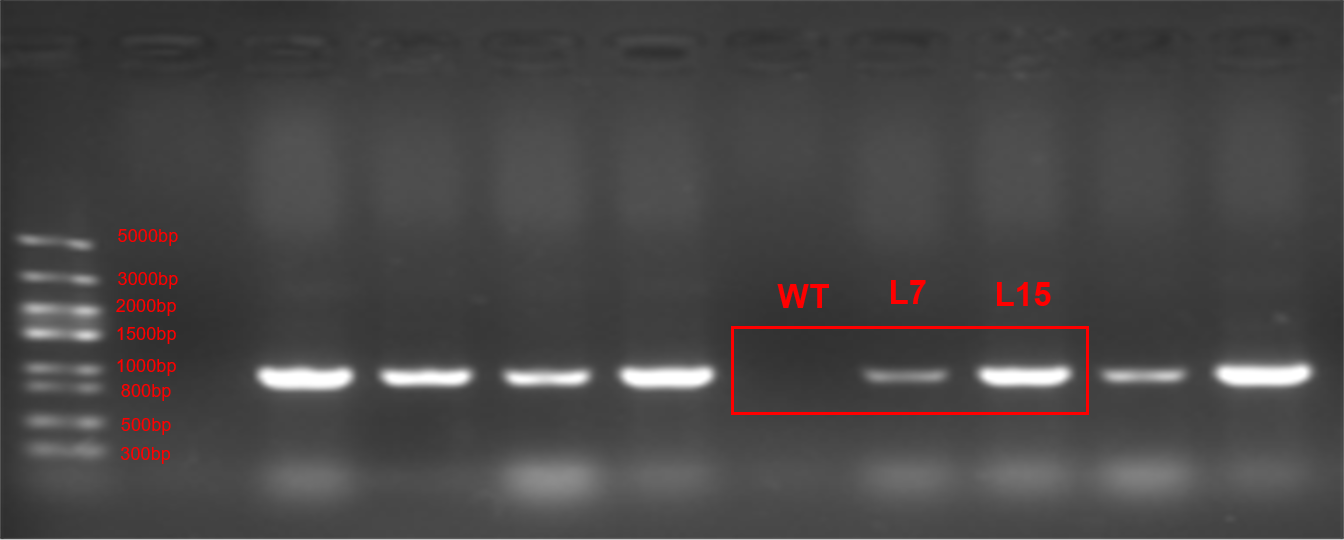


TaSPL6B-F：GGTGTGCTGTGACCAAAATGCTG

TaSPL6B-R：AACTTCAGGGTCAGCTTGC
